# Maternal mortality ratios in 2852 Chinese counties, 1996–2015, and achievement of Millennium Development Goal 5 in China: a subnational analysis of the Global Burden of Disease Study 2016

**DOI:** 10.1016/S0140-6736(18)31712-4

**Published:** 2019-01-19

**Authors:** Juan Liang, Xiaohong Li, Chuyun Kang, Yanping Wang, Xie Rachel Kulikoff, Matthew M Coates, Marie Ng, Shusheng Luo, Yi Mu, Xiaodong Wang, Rong Zhou, Xinghui Liu, Yali Zhang, Yubo Zhou, Maigeng Zhou, Qi Li, Zheng Liu, Li Dai, Mingrong Li, Yiyi Zhang, Kui Deng, Xinying Zeng, Changfei Deng, Ling Yi, Jun Zhu, Christopher J L Murray, Haidong Wang

**Affiliations:** a National Office for Maternal and Child Health Surveillance of China, West China Second University Hospital, Sichuan University, Chengdu, Sichuan, China; b Department of Obstetrics, West China Second University Hospital, Sichuan University, Chengdu, Sichuan, China; c National Center for Birth Defect Surveillance of China, West China Second University Hospital, Sichuan University, Chengdu, Sichuan, China; d Office for National Maternal and Child Health Statistics of China, School of Public Health, Peking University, Beijing, China; e Institute for Health Metrics and Evaluation, University of Washington, Seattle, WA, USA; f Department of Global Health and Social Medicine, Harvard University, Boston, MA, USA; g IBM Watson Health, San Jose, CA, USA; h Department of Maternal and Child Health, School of Public Health, Peking University, Beijing, China; i National Center for Chronic and Noncommunicable Disease Control and Prevention, Chinese Center for Disease Control and Prevention, Beijing, China; j Institute of Reproductive and Child Health, Ministry of Health Key Laboratory of Reproductive Health, Peking University, Beijing, China; k Key Laboratory of Birth Defects and Related Diseases of Women and Children (Sichuan University), Ministry of Education, Chengdu, Sichuan, China

## Abstract

**Background:**

As one of only a handful of countries that have achieved both Millennium Development Goals (MDGs) 4 and 5, China has substantially lowered maternal mortality in the past two decades. Little is known, however, about the levels and trends of maternal mortality at the county level in China.

**Methods:**

Using a national registration system of maternal mortality at the county level, we estimated the maternal mortality ratios for 2852 counties in China between 1996 and 2015. We used a state-of-the-art Bayesian small-area estimation hierarchical model with latent Gaussian layers to account for space and time correlations among neighbouring counties. Estimates at the county level were then scaled to be consistent with country-level estimates of maternal mortality for China, which were separately estimated from multiple data sources. We also assessed maternal mortality ratios among ethnic minorities in China and computed Gini coefficients of inequality of maternal mortality ratios at the country and provincial levels.

**Findings:**

China as a country has experienced fast decline in maternal mortality ratios, from 108·7 per 100 000 livebirths in 1996 to 21·8 per 100 000 livebirths in 2015, with an annualised rate of decline of 8·5% per year, which is much faster than the target pace in MDG 5. However, we found substantial heterogeneity in levels and trends at the county level. In 1996, the range of maternal mortality ratios by county was 16·8 per 100 000 livebirths in Shantou, Guangdong, to 3510·3 per 100 000 livebirths in Zanda County, Tibet. Almost all counties showed remarkable decline in maternal mortality ratios in the two decades regardless of those in 1996. The annualised rate of decline across counties from 1996 to 2015 ranges from 4·4% to 12·9%, and 2838 (99·5%) of the 2852 counties had achieved the MDG 5 pace of decline. Decline accelerated between 2005 and 2015 compared with between 1996 and 2005. In 2015, the lowest county-level maternal mortality ratio was 3·4 per 100 000 livebirths in Nanhu District, Zhejiang Province. The highest was still in Zanda County, Tibet, but the fall to 830·5 per 100 000 livebirths was only 76·3%. 26 ethnic groups had population majorities in at least one county in China, and all had achieved declines in maternal mortality ratios in line with the pace of MDG 5. Intercounty Gini coefficients for maternal mortality ratio have declined at the national level in China, indicating improved equality, whereas trends in inequality at the provincial level varied.

**Interpretation:**

In the past two decades, maternal mortality ratios have reduced rapidly and universally across China at the county level. Fast improvement in maternal mortality ratios is possible even in less economically developed places with resource constraints. This finding has important implications for improving maternal mortality ratios in developing countries in the Sustainable Development Goal era.

**Funding:**

National Health and Family Planning Commission of the People's Republic of China, China Medical Board, WHO, University of Washington Center for Demography and Economics of Aging, Bill & Melinda Gates Foundation.

## Introduction

Over the past two decades, China as a nation has made impressive progress in improving maternal and child health. Mortality among children younger than 5 years has declined from 54·1 per 1000 livebirths in 1990 to about 12·5 per 1000 livebirths in 2015, giving an annualised rate of decline of 5·9% per year.^1^ This percentage far exceeds the annual 4·4% decline required to achieve Millennium Development Goal (MDG) 4.^1^ Over the same period, China has pushed down the maternal mortality ratio at an annualised rate of 6·5% per year, one of the fastest decreases in the world. The national maternal mortality ratio fell from 111·0 per 100 000 livebirths in 1990 to 89·4 per 100 000 livebirths in 2000, and to 21·8 per 100 000 livebirths in 2015.^2^

**Research in context**

**Evidence before this study**

We searched PubMed and the China National Knowledge Infrastructure database with the terms “maternal mortality”, “county”, “district”, and “China”. As one of a handful of countries that have achieved Millennium Development Goals (MDGs) 4 and 5, China has made substantial progress in improving maternal and child health in the past two decades. Various studies reported in English and Chinese literature have documented the rapid decline in maternal mortality in China since the early 1990s, but most provide estimates of maternal mortality only at national or regional levels. Few have assessed maternal mortality at the provincial level and, to our knowledge, no study has provided systematic assessment of maternal mortality ratio at the county level in China.

**Added value of this study**

We used data including information on maternal mortality and associated key factors, such as numbers of livebirths and hospital delivery rates, for all counties in China between 1996 and 2015. A Bayesian hierarchical small-area estimation model with latent Gaussian layers was used in our analysis that accounted for space and time correlations in maternal mortality ratios among neighbouring counties. The county-level estimates from the small-area estimation model were scaled up to national-level maternal mortality ratio estimates from the Global Burden of Diseases, Injuries, and Risk Factors Study 2016 to account for under-reporting in China's national Annual Report System on Maternal and Child Health. The modelling effort in our study produced comprehensive and internally comparable estimates of maternal mortality ratios for 2852 counties in China for the past two decades. We found rapid decline in maternal mortality ratios in almost all counties since 1996, but also substantial heterogeneity in the amount of decline and in trends across counties in different provinces and regions. Our findings provide a road map for local health authorities to target interventions to further improve maternal mortality ratio in regions and areas where ratios are high. Additionally, they offer a launch pad for researchers to study factors associated with high maternal mortality ratios and to identify effective intervention programmes. Knowledge gained from such analyses will be helpful for other countries that are trying to achieve the Sustainable Development Goal on improving maternal health.

**Implications of all the available evidence**

Although China has made important advances in lowering maternal mortality at the national level and has achieved MDG 5 by lowering the maternal mortality ratio by 75% from 1990 to 2015, the degree and trends for maternal mortality remain widely heterogeneous at the county level. Regardless of the level in 1996, the pace of decline in maternal mortality ratios has at least matched the target of MDG 5 in most counties. More than 461 counties, however, still had maternal mortality ratios twice as high as the national average in 2015. Thus, despite improving maternal health in China, much needs to be done to close the gaps between counties.

National progress might mask variation across counties within China. Analyses of child mortality have shown substantial variation at the county level in mortality among children younger than 5 years and in the pace of progress.^1^ Given the sensitivity of maternal mortality to the availability of quality prenatal, intrapartum, and postpartum care, progress on reducing maternal mortality might have varied as much across counties. Although as a nation China has already achieved the Sustainable Development Goal (SDG) target of reducing the rate of maternal deaths to 70 per 100 000 livebirths, maternal mortality ratios have not been assessed at the county level.^3^, ^4^, ^5^, ^6^, ^7^, ^8^ Analyses of levels and trends in maternal mortality ratio at the local level would help to identify successes and communities that might need extra resources to accelerate progress. As China's effort to improve population health among its partner countries under the Belt and Road Initiative expands, performance among the Chinese counties will be important to study, particularly in those with lower economic levels. The findings could guide the Chinese government and public health policy makers in other countries to devise effective plans for improving maternal and child health.

The purpose of this study was to use all available data sources to estimate the maternal mortality ratio for 2852 Chinese counties from 1996 to 2015. Because of the relatively small number of livebirths and the smaller number of observed maternal deaths in individual counties in any given year, we expected great fluctuation of observed maternal mortality ratios at the county level. Thus, we aimed to estimate the underlying risk of maternal mortality that is affected by relevant key determinants, such as maternal education and level of economic development. We aimed to use the findings to identify inequalities in maternal mortality ratios by level and speed of progress in different locations and to assess the relationships between levels and speed of progress in improving maternal mortality ratios in counties in which large fractions of the population belonged to ethnic minorities.

## Methods

### Data

We used the national Annual Report System on Maternal and Child Health (ARMCH) as our main data source. Established in the early 1980s, the ARMCH covers all counties and county-equivalent administrative units, such as districts within cities and special economic zones. Tabulated data are provided on key maternal and child health metrics, including livebirths, deaths due to maternal causes, and deaths in children younger than 5 years, and key health-service-related indicators, such as management during pregnancy, pregnancy wellness check-ups, health check-ups among children younger than 7 years, in-hospital delivery rates, and neonatal check-up rates. We also collected county-level data on gross domestic product per capita from provincial-level and county-level statistical reports. For consistency, county-level estimates are scaled to the provincial level used in the Global Burden of Diseases, Injuries, and Risk Factors Study (GBD) 2016 by application of a constant scaling factor to all county-level gross domestic product estimates.

We computed lag-distributed income at the county level, which is the weighted average of gross domestic product per capita in a 10-year period. Missing values at the county level are imputed with the standard missing data method.^9^

In 2015, there were 2856 county-level administrative units in China. Four counties were excluded from our analysis: three belong to the newly formed Sansha City in the South China Sea, with a combined civilian population of less than 2500, and the other is Jingmen County from Fujian Province, which did not report any data to the ARMCH system. Given the changes in administrative boundaries over the past two decades among the remaining 2852 counties, we collapsed counties into 2732 analytical county units for our analysis.

### Small-area estimation model

Estimating health indicators for small areas such as counties is intrinsically challenging, especially because maternal mortality is a rare event and in China the total fertility rate is low. Previous studies of mortality among children younger than 5 years or other age-specific mortality at the county level in China and the USA have used Bayesian estimation methods with covariates, such as income and education, to inform the levels and trends of the indicator of interest and to borrow strength over time and space.^9^, ^10^, ^11^

We used a Bayesian spatially explicit mixed-effects regression model fitted with the INLA program in R version 3.4.1.^12^ We estimated values with the following equation, in which a negative binomial distribution was specified for the maternal deaths at the county level with the corresponding livebirths as offset.

$$\text{In}(D_{c,t})=\text{In}(births_{c,t})+\beta_{0}+(\beta_{1}\times LDI)+(\beta_{2}\times education)+(\beta_{3}\times year)+{ɛ}_{c}+{ɛ}_{p,s}+S_{c}+t_{t}$$

In(*D*_c,t_) indicates the number of maternal deaths in logarithmic scale by county and year, *S*_c_ represents a spatial intrinsic conditional autoregressive random effect, ε_c_, ε_p,s_, and *t*_t_ are independent and identically distributed random effects on country, province strata, and year, respectively, and the covariates LDI (lag-distributed income) and education are county and year specific.

To strengthen and improve robustness of estimates at the county level, we added spatial random effects for each county and the correlations between them. One county random effect has an intrinsic conditional autoregressive prior, implying that the random effect for a specific county is normally distributed around the mean of the random effects in neighbouring counties. This specification was used to achieve spatial smoothing. A second county-level random effect has an independent and identically distributed prior. Our combined model allowed for spatially structured and unstructured variations. We also added a random effect by province and urban or rural strata to capture the intrinsic difference between rural and urban areas, and the differential levels of social and economic development among the 31 provinces in mainland China. 1000 draws of each In(*D*_c,t_) were taken from the posterior distribution to calculate draw-level maternal mortality ratios for each county by year.

### Scaling estimates to national levels

To ensure that estimates of maternal mortality ratios at the county level were consistent over time, we scaled the county-level estimated maternal mortality ratios to the national level estimates in GBD 2016 with the method described for gross domestic product. This scaling step was included to account for incomplete registration of maternal deaths and reported livebirths in the ARMCH system at the county level. We used the national-level estimates from GBD 2016 because a cause-of-death ensemble model was used that estimates maternal mortality by combining model predictions from selected statistical models based on various specifications of covariates and functional forms. The cause-of-death ensemble model also draws information from other countries.^13^ The key data for estimation of the national maternal mortality ratio were taken from the National Maternal and Child Health Surveillance System, which originated in 1989. In 1996, after integration with surveillance systems that captured data on mortality among children younger than 5 years and on birth defects, a new nationally representative sample was selected that included over 80 million people in 176 counties and districts in China. In 2006, further adjustments were made to the sample framework, and the surveillance system was expanded to cover 140 million people in 334 counties. For our analysis, we applied a yearly scaling factor to county-level estimates to achieve weighted average county-level maternal mortality ratios after scaling that matched the national-level ratios in GBD 2016.

### Analysis by ethnic group

We aggregated estimated county-level maternal mortality ratios by ethnic groups. As separate data by ethnicity are not available in ARMCH, we grouped counties in which more than 50% of the population consisted of a specific ethnic group.

### Inequality analysis

We investigated inequality of maternal mortality ratios at the county level by computing Gini coefficients of maternal mortality ratio, weighted by the number of annual livebirths at the county level and unweighted.^1^ We used the following equation.

$$Gini=\frac{\sum_{i=1}^{2852}\sum_{j=1}^{2852}(|x_{i}-x_{j}|)p_{i}p_{j}}{2{\left(\sum_{j=1}^{N}p_{j}\right)}^{2}\mu }$$

In this equation, *x* is maternal mortality ratio at the county level, *p* is the number of livebirths at the county level, and *i* and *j* indicate counties. Gini coefficients may be interpreted as the average relative differences between all pairs of counties.

### Role of the funding source

The funder of the study had no role in the study design, data collection, data analysis, data interpretation or the writing of the report. The corresponding author had full access to all the data in the study and had the final responsibility for the decision to submit for publication.

## Results

From 1996 to 2000, the maternal mortality ratio in China declined by about 18%, from 108·73 per 100 000 livebirths in 1996 to 89·4 per 100 000 livebirths in 2000, which would roughly equate to an annualised rate of 4·9%. Since 2000, decline at the national level has accelerated, falling at an annualised rate of 9·4% up to 2015. Progress at the county level, however, has been heterogeneous.

In 1996, the subnational-level maternal mortality ratio ranged from 16·8 (95% uncertainty interval 10·5–24·9) per 100 000 livebirths in Haojiang District, Shantou, Guangdong Province, to 3510·3 (1857·8–5885·0) per 100 000 livebirths in Zanda County, Tibet (table 1). Among the 195 countries for which GBD provides national estimates, maternal mortality ratio ranged from 3·7 per 100 000 in Iceland to 1023·7 per 100 000 livebirths in Somalia. 31 counties in China had maternal mortality ratios greater than 1023·7 per 100 000 livebirths. In the same year, 55 counties in China had maternal mortality ratios lower than 30 per 100 000 livebirths, which are similar to those in developed countries such as Finland and Sweden in 1996. 52 of these 55 counties were concentrated in historically affluent provinces of Jiangsu, Zhejiang, Guangdong, and Shanghai. Haojiang District in the City of Shantou, Guangdong Province, had the lowest county-level maternal mortality ratio in China in 1996, although at 16·8 per 100 000 livebirths, it is more than 354% higher than the lowest national-level maternal mortality ratio observed across countries that year.

**Table 1:** Maternal mortality ratios and annualised rates of decline from 1996 to 2015 in the top and bottom performing counties in 2015

	**Province**	**County or district**	**Maternal mortality ratio (deaths per 100 000 livebirths)**		**Annualised rate of decline in maternal mortality ratio 1996–2015 (%)**
			1996	2015	
**Highest ranking**					
1	Zhejiang	Nanhu District	17·0	3·4	8·4%
2	Zhejiang	Pinghu City	18·7	3·5	8·9%
3	Guangdong	Haojiang District	16·8	3·6	8·1%
4	Guangdong	Longhu District	18·2	3·9	8·1%
5	Guangdong	Jinping District	18·8	4·1	8·0%
6	Zhejiang	Haiyan County	20·3	4·1	8·5%
7	Zhejiang	Jiashan County	23·2	4·4	8·8%
8	Zhejiang	Jiangdong District	28·1	4·7	9·4%
9	Zhejiang	Tong County City	23·9	4·7	8·5%
10	Zhejiang	Yuecheng District	24·4	4·8	8·6%
11	Zhejiang	Keqiao District	27·7	4·8	9·3%
12	Jiangsu	Kunshan City	27·2	4·8	9·1%
13	Shanghai	Jing'an District	26·0	4·9	8·8%
14	Zhejiang	Wenling City	23·7	5·0	8·2%
15	Guangdong	Nanshan District	27·1	5·0	8·9%
16	Zhejiang	Changxing County	25·9	5·1	8·6%
17	Shanghai	Changning District	25·1	5·1	8·4%
18	Zhejiang	Haining City	27·3	5·2	8·7%
19	Shanghai	Xuhui District	22·2	5·2	7·6%
20	Zhejiang	Binjiang District	61·2	5·3	12·9%
**Lowest ranking**					
2833	Tibet	Banbar County	1255·0	295·7	7·6%
2834	Tibet	Nyainrong County	1278·9	298·5	7·7%
2835	Tibet	Gamba County	1329·4	298·9	7·9%
2836	Tibet	Amdo County	1581·7	307·2	8·6%
2837	Tibet	Dinggyee County	1597·3	310·1	8·6%
2838	Tibet	Saga County	2419·1	313·2	10·8%
2839	Tibet	Shuanghu County	1689·5	330·6	8·6%
2840	Tibet	Ngamring County	1657·3	338·8	8·4%
2841	Tibet	Geerzee County	1635·0	340·5	8·3%
2842	Tibet	Tingri County	1945·6	342·4	9·1%
2843	Tibet	Nyima County	1771·2	346·6	8·6%
2844	Tibet	Coqeen County	2464·3	373·7	9·9%
2845	Tibet	Bangoin County	1678·7	375·5	7·9%
2846	Tibet	Nyalam County	1572·5	383·0	7·4%
2847	Tibet	Rutog County	1517·1	424·1	6·7%
2848	Tibet	Gar County	2212·0	461·2	8·3%
2849	Tibet	Zhongba County	3066·1	473·5	9·8%
2850	Tibet	Burang County	2932·5	575·7	8·6%
2851	Tibet	Gee'gyai County	2688·3	667·0	7·3%
2852	Tibet	Zanda County	3510·3	830·5	7·6%

**Figure 1:**
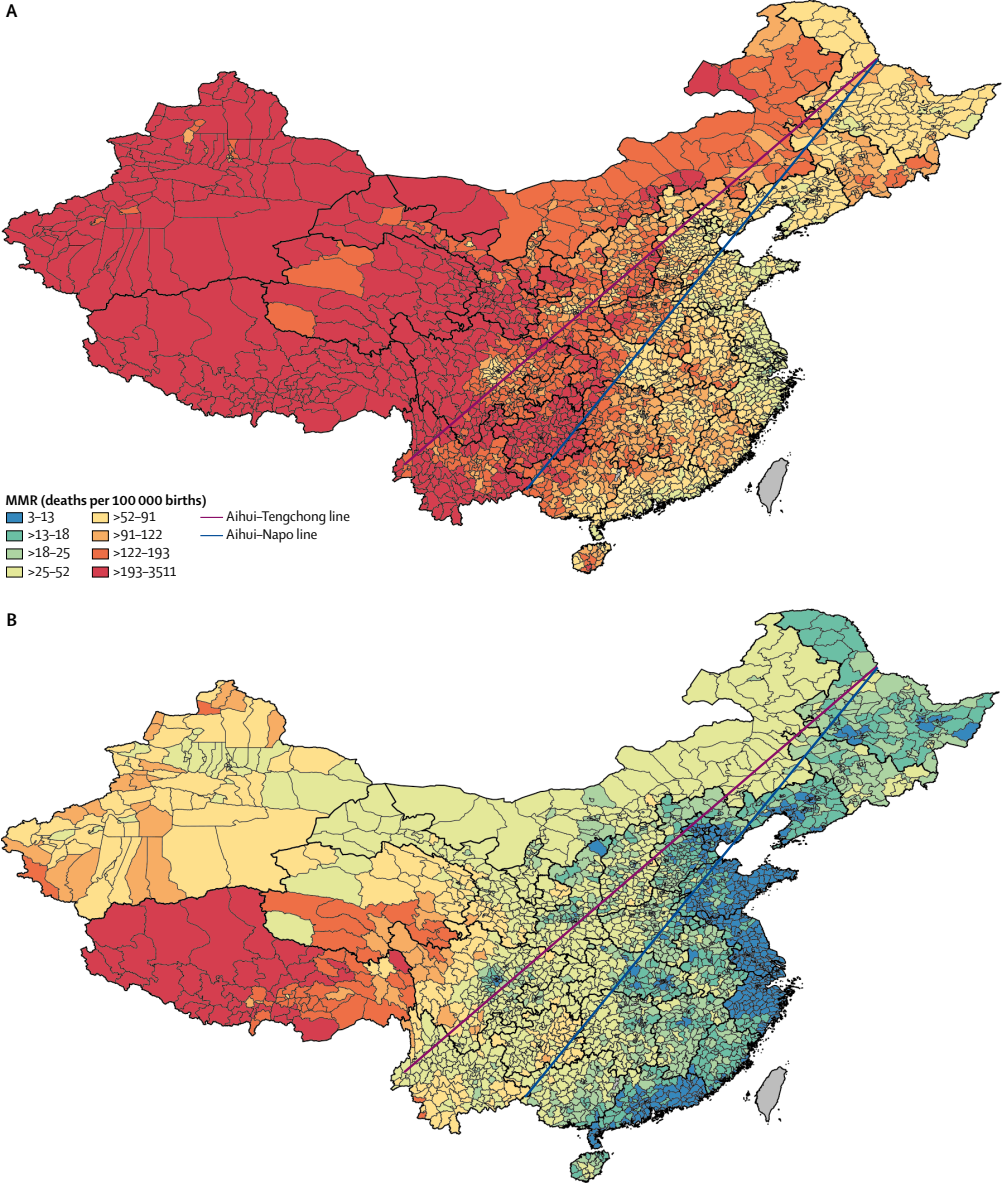
County-level maternal mortality ratios in mainland China in 1996 and 2015 (A) 1996. (B) 2015. MMR=maternal mortality ratio.

Counties with lower maternal mortality ratios (<52 per 100 000 livebirths) were concentrated mostly in the east and southeast parts of China (figure 1A). This regional distribution pattern closely matches the geographical divide of China, represented by the Aihui-Tengchong line established by Huanyong Hu in 1935 (figure 1), which was based on population density and economy (agriculture *vs* hunting and gathering). Although simple, it provides a fairly accurate point of reference for different levels of urbanisation in the west and east of China. The distribution remained similar in 2015 (figure 1B) except for in three provinces (Guizhou, Sichuan, and Yunnan) where county-level maternal mortality ratios were relatively high. If the southwest point of the geographical dividing line is moved from Tengchong in Yunnan Province to Napo County in Guangxi Province (figure 1), an even more obvious pattern of maternal health emerges, and reflects the pattern of mortality among children younger than 5 years at the county level.^1^

**Figure 2:**
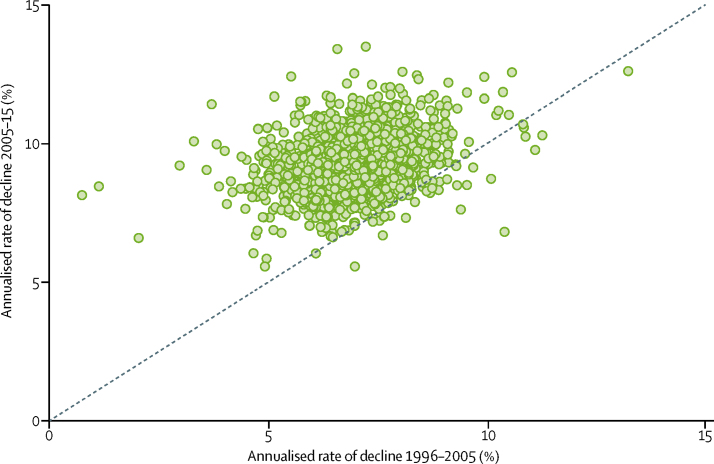
Annualised rate of decline in maternal mortality ratios from 1996 to 2005, and 2005 to 2015

Regardless of values in 1996, almost all counties in China showed rapid decline in maternal mortality ratio by 2015. The annualised rate of decline between 1996 and 2015 ranged from 4·4% (95% CI 2·8–6·1) in Chongyang County in Hubei Province to 12·9% (11·2–14·6) in Binjiang District in Zhejiang Province (figure 2). Of note, the correlation between maternal mortality ratios in 1996 and the annualised rate of decline in maternal mortality ratios to 2015 is minimal (*r*=0·087), which is not surprising given that 99·8% of the counties in China have achieved the target annualised rate of decline in MDG 5. Also, the ranges and the mean values for annualised rates of decline were very similar for counties in the top and bottom halves of the maternal mortality ratio range in 1996.

Among the 31 counties that had maternal mortality ratios higher than the 1023·7 per 100 000 livebirths seen in Somalia in 1996, the annualised rates of decline ranged from 6·7% to 10·8%, with a mean rate of 8·5%, which was faster than the national mean rate of decline in China of 8·2% per year. In only six counties were rates of decline in maternal mortality ratio slower than the MDG 5 target pace of 5·5% per year: Xiangyang District in Heilongjiang Province (5·4%); Zhaoling District in Henan Province (5·4%); Chongyang County and Sui County in Hubei Province (4·4% and 5·3%, respectively); Jiali County in Tibet (5·0%); and Hongsibao District in Ningxia Hui Autonomous Region (4·6%).

**Figure 3:**
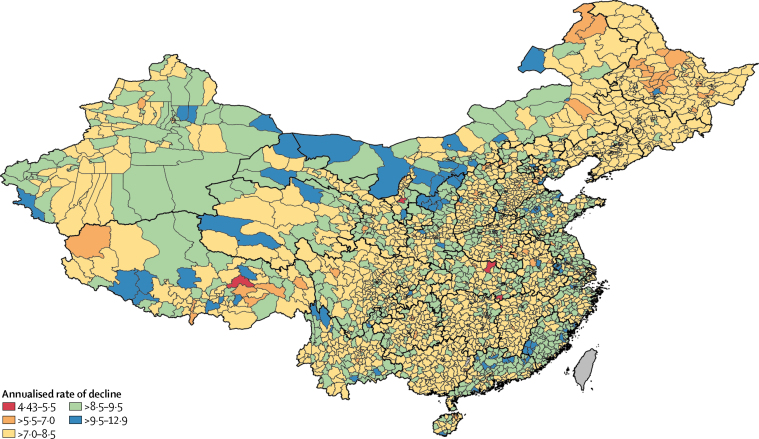
County-level annualised rates of decline in maternal mortality ratio from 1996 to 2015 in mainland China

Since the implementation of the programme Reducing Maternal Mortality and Eliminating Neonatal Tetanus, also known as the Reducing and Eliminating programme, in 2000, the rates of decline in maternal mortality ratio at the county level have accelerated in most of the counties in China. Because the rollout of the programme was gradual, we have analysed rates of decline from 1996 to 2005 and from 2005 to 2015. The annualised rate of decline in maternal mortality ratio ranged from 0·7% to 13·2% from 1996 to 2005, giving a mean annualised rate of decline of 7·0%. The annualised rate of decline improved year on year from 2005 to 2015. The mean county-level annualised rate of decline for this period was 9·3%, representing a 35·1% increase over the mean annualised rate of decline from the previous decade, with acceleration in decline seen in 2812 (98·6%) counties (figure 3). When comparing the rate of decline in maternal mortality ratio for 1996–2005 and 2005–15, in 2394 (83·9%) counties the annualised decline increased by more than 20% in the later decade. Among them, 457 counties (19·1%) saw improvements of more than 50%. On average, the annualised rate of decline in maternal mortality ratio at the county level increased by 36·2% in the period 2005–15, compared with that in the previous decade. 24 counties at least doubled their rates of decline in 2005–15, with the greatest improvement being seen in Hongsibao District in the City of Wuzhong, Ningxia Hui Autonomous Region (increase 996·2% from 0·7%, 95% CI −1·6 to 3·1 in 1996–2005, to 8·2%, 5·1 to 11·1 in 2005–15). The other 23 counties with increases in the annualised rate of decline greater than 100% were in 15 provinces with varying levels of economic development. Only 39 of 2852 counties showed a slowdown in improvement of maternal mortality ratio from 2005 to 2015.

The rapid decline in maternal mortality across China since 1996 has led to much greater reductions by 2015, when maternal mortality ratios ranged from 3·4 (95% CI 1·7–6·1) per 100 000 livebirths in Nanhu Cistrict, Zhejiang Province, to 830·5 (427·2–1494·7) per 100 000 livebirths in Zanda County in Tibet. Of note, however, in Zanda County only one maternal death was reported with 104 livebirths in the ARMCH system. The lowest county-level maternal mortality ratio in 2015 is similar to those in the most developed countries, including Sweden (3·3 per 100 000 livebirths), Finland (3·4 per 100 000 livebirths), Norway (3·6 per 100 000 livebirths), Italy (3·7 per 100 000 livebirths), and Austria (4·0 per 100 000 livebirths). Among the 195 countries included in GBD 2016, only 24 have maternal mortality ratios lower than 6·0 per 100 000 livebirths. In China, 48 counties have maternal mortality ratios below this level. Additionally, 93% of all counties in China have maternal mortality ratios at or below the targeted SDG level at 70 per 100 000 livebirths.

With 56 ethnic groups in China, cultural practices and socioeconomic conditions are highly heterogeneous. We were able to capture maternal mortality ratio levels and trends for 26 ethnic groups that represented the majority of the population in at least one county, including the Han ethnic group. More than 50% of the population belonged to one ethnic minority that was not Han Chinese in 282 counties. These people represented about 3·6% of the total population in China. Most of the ethnic minority counties were located in regions with economic development below the average for China, although the association between economic development and reduction in maternal mortality ratio was very weak in 1996–2015. Han Chinese were the majority of the population in the greatest number of counties and had the second lowest maternal mortality ratio in 2015 (table 2), but this rate was only about 0·3 per 100 000 livebirths higher than the counties in which the Manchu ethnic group was the majority, meaning that the annualised rate of decline in maternal mortality ratio was average (table 2). Four ethnic groups, Tajik, Dongxiang, Kirgiz, and Hani, showed annualised rates of decline in maternal mortality ratio greater than 9% from 1996 to 2015 (table 2). The 25 ethnic minority groups other than the Han Chinese group had rates of decline greater than the MDG 5 target rate.

**Table 2:** Livebirth-weighted averages and annualised rates of decline in maternal mortality ratios in counties where at least 50% of the population consists of a specific ethnic group in 1996–2015

	**Maternal mortality ratio (deaths per 100 000 livebirths)**		**Annualised rate of decline in maternal mortality ratio 1996–2015 (%)**	**Number of counties with an ethnic group comprising ≥50% population**	**Total county population (thousands)**
	1996	2015			
Tajik	914·2	141·5	9·8%	1	30·5
Dongxiang	275·5	47·1	9·3%	1	256·8
Kirgiz	527·1	94·8	9·2%	2	76·3
Hani	258·2	46·5	9·1%	3	831·8
Lisu	438·3	82·6	8·8%	3	406·1
Kazak	459·1	87·0	8·8%	6	385·2
Moinba	800·4	153·4	8·7%	1	9·7
Salar	258·7	50·9	8·6%	1	104·5
Zhuang	171·5	32·3	8·4%	5	2070·8
Li	140·0	28·7	8·4%	2	265·3
Tujia	197·9	40·8	8·4%	15	6090·3
Bai	164·0	33·2	8·3%	5	1448·1
Uygur	352·2	72·5	8·3%	34	7698·1
Han	91·9	18·2	8·3%	2143	1 017 660·0
Miao	236·8	47·8	8·3%	11	2837·6
Qiang	193·7	41·3	8·3%	2	263·9
Bouyei	250·6	53·0	8·2%	5	1271·4
Tibetan	649·3	152·4	8·2%	125	5232·2
Shui	250·9	54·2	8·1%	1	297·4
Manchu	79·3	17·9	8·0%	18	6816·3
Mongolian	161·5	37·0	8·0%	9	1573·0
Yi	311·2	74·2	8·0%	11	1989·5
Dong	216·0	45·0	8·0%	6	1920·5
Hui	221·1	47·1	7·9%	7	1621·6
Yao	129·9	29·2	7·8%	4	1078·8
Korean	104·1	23·2	7·5%	4	1041·5

**Figure 4:**
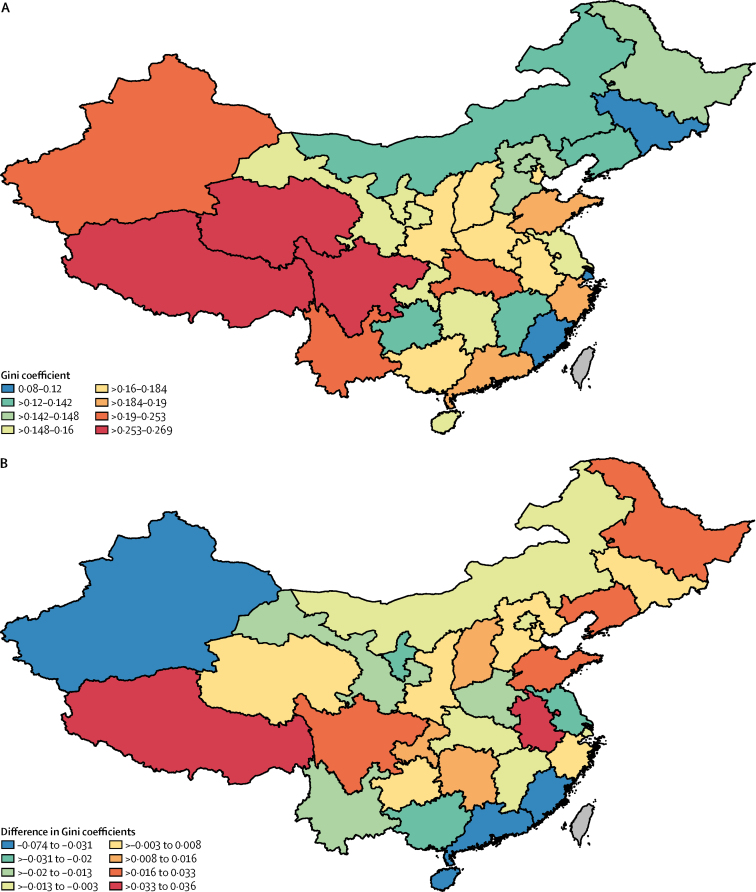
Inequality of maternal mortality ratios at the province level in mainland China Measurements were calculated as Gini coefficients. (A) 2015. (B) Changes from 1996 to 2015.

With swift economic growth in China over the past three decades and heterogeneous rates of change in income per capita at the county level since 1996,^14^ income equality has been a concern. However, the diversity in economic development has not been translated into inequality in maternal mortality ratio at the county level (figure 4). From 1996 to 2015, the Gini coefficient of maternal mortality ratio increased only slightly, from about 0·63 to 0·65, when not weighted by the number of livebirths. With weighting, the Gini coefficient is 0·35 for 1996 and 2015. The intercounty inequality of maternal mortality ratio in China, therefore, seems to have remained stable over the past two decades. By contrast, the intercountry inequality of maternal mortality ratio worldwide has increased from 0·597 to 0·643 in the same period when weighted by the number of livebirths.

Inequalities within provinces are much smaller than across provinces. The livebirth-weighted intercounty Gini coefficient at the province level is about half of that at the county level. However, the trend of inequality at the province level has varied over the past two decades (figure 4). 17 (55%) of the 31 provinces have seen decreasing inequality in maternal mortality ratio over the past two decades. Among them, Hainan and Fujian have had decreases of more than 30% from 1996 to 2015. Five other provinces—Ningxia Hui Autonomous Region, Jiangsu, Guangdong, Guangxi, and Xinjiang—have had Gini coefficient declines of at least 10%. Overall economic development in these seven provinces has varied substantially. For instance, Guangdong Province is one of the most economically developed regions in China, but Ningxia Hui Autonomous Region has among the lowest income per capita in China. Conversely, seven provinces experienced increases in Gini coefficients of more than 10% from 1996 to 2015. Among them, Anhui and Liaoning had increases of more than 20·0% (28·5% and 24·5%, respectively), and in the remaining five (Heilongjiang, Shandong, Chongqing, Sichuan, and Tibet) the range of increases was 10·0–16·0%. Again, increase in inequality in maternal mortality ratio at the provincial level does not seem to correlate with levels of economic development. While Tibet and Anhui are the poorest provinces in China, Zhejiang and Chongqing, which had Gini coefficient increases of 3·9% and 10·0%, respectively, are among the richest provinces by the International Monetary Fund's most recent measurement.

## Discussion

Even in China, where there are more than 17 million livebirths each year,^15^ maternal mortality is low. We were able to use available data sources to estimate maternal mortality ratios for every district and county level over a 20-year period. Our estimated maternal mortality ratio represents the underlying risk of dying from maternal causes, which should differ from the observed maternal mortality ratios from a reported source, such as ARMCH. We found substantial variation in maternal mortality ratio across counties, but all showed declines in maternal mortality greater than the MDG 5 target of 5% per year. Despite this remarkable progress, 191 (6·7%) counties remained above the SDG target level of 70 per 100 000 livebirths.

It is natural to assume that improved economic conditions contribute substantially to decline in maternal mortality ratio. Nevertheless, income per capita at the county level in 1996 in China explained only about 10% of the variation in the annualised rate of decline in maternal mortality ratios over the next two decades. Furthermore, the improvement in income from 1996 to 2015 explains only about 18% of the changes in maternal mortality ratio. Low fertility rates might partly contribute to low maternal mortality ratios, but the declines seem more likely to be related to interventions introduced under the national Reducing Maternal Mortality and Eliminating Neonatal Tetanus programme from 2000. After the setting of the MDGs, in view of the inequality of maternal mortality between economically rich and deprived regions, the central government of China established this national programme to lower maternal mortality in rural regions. It was funded by the State Council Committee for Women and Children, the Ministry of Finance, and the Ministry of Health. While it was initially rolled out to 1000 counties in the midwestern regions of China, it had become a national programme by 2009.

The Reducing Maternal Mortality and Eliminating Neonatal Tetanus programme should be considered as one of the most ambitious national public health interventions in China and globally. It has six major objectives: improve obstetric health care in hospitals at the county, township, and village levels; set up obstetric emergency centres in all 2852 counties of China; develop a so-called green channel service for obstetric emergencies at hospitals at the county level to ensure ambulances and medical personnel are available all day and night; improve the percentage of in-hospital deliveries by reducing or waiving costs, especially for women in rural areas and with low incomes; improve health education through community health workers; and strengthen support and supervision of obstetric health care in hospitals at all levels. In 2812 (98·6%) of 2852 counties, the decline in maternal mortality ratios has accelerated since 2005. When comparing the rate of decline in maternal mortality ratio for 1996–2005 and 2005–15 to account for the gradual rollout of the Reducing and Eliminating programme, 2394 (83·9%) counties showed increased annualised declines in maternal mortality ratio, by 20% more in the most recent decade. Among them, 457 (19·1%) counties showed improvements in the annualised rates of decline by over 50%. While this study does not prove that the remarkable rates of progress are related to any particular government policy or programme, it seems credible that these policies could account for the widespread acceleration in declining maternal mortality ratios and the pace of progress that far exceeds what might be explained by economic growth and reduction in fertility. China's success in lowering the maternal mortality ratio provides a case study that could help other countries with high ratios reach the SDG goal before 2030.

Also of note is that China has one of the highest rates of caesarean sections in the world. A WHO study from 2010 shows that 46% of the babies born in China between 2004 and 2008 were delivered by caesarean section rather than by vaginal birth.^16^ In addition, along with the general economic development in the past three decades, systematic improvements in access to health care, road conditions, and other socioeconomic factors might all have contributed to the improvements in maternal and child health, and the improvement in maternal mortality.

We could not estimate how many counties have achieved MDG 5 because the ARMCH data are only available from 1996, but the estimated annualised rate of decline in maternal mortality ratio between 1996 and 2015 shows that only six counties had not achieved the MDG 5 target rate. Nevertheless, 191 counties had not met the the SDG target in 2015, and the highest maternal mortality ratio was 830·5 per 100 000 livebirths. The central and local governments need to make a concerted effort to help these counties achieve the SDG target by 2030. More in-depth case studies will be needed to devise appropriate interventions for these counties. Previous studies have pointed out cultural practices and low percentages of in-hospital delivery as major factors behind high maternal mortality ratios in the southwest region of China,^3^, ^8^ where a high percentage of the population consists of ethnic minorities. Tough natural environments, difficulty in accessing convenient means of transportation, and weaker health services at the community level might also contribute to the high maternal mortality ratios.

In 2007, the 17th Chinese Communist Party's National Congress decided to build a national public health system to achieve universal basic coverage for all Chinese citizens. Providing free maternal health care and management at community health centres was one of the 12 programmes established in 2009. At the same time, in-hospital delivery based on a cash-for-service model in rural areas was expanded to all counties in China. From 2010 to 2015, the in-hospital delivery rate increased from 96·3% to 99·7%. In the same period, substantial efforts were also devoted to providing systematic care for mothers from before to after birth, fetuses before and during delivery, and neonates for up to 42 days after delivery, and the percentage of pregnancies being managed in this way increased from 80·9% to 91·5%.^15^ While such improvement is impressive, 461 counties still had maternal mortality ratios at least twice as high as the national level in 2015. Further improvements in access to and quality of health care, especially in rural areas, are essential. More specifically, increasing the number of well educated and highly trained midwives will be an important factor in improving maternal health in China in the coming decades. Despite over 17 million livebirths every year, in 2007 there were only about 35 000 midwives in China,^17^ or about 0·03 midwives per 1000 population.^18^ This number is extremely low compared with the numbers in high-income nations, such as the UK (0·63), and China's peers in Asia, including Vietnam (0·09), Japan (0·16), South Korea (0·19), Cambodia (0·23), and Mongolia (0·24). In addition, no independent education or training for midwives has been provided in China since the late 1960s. In Chinese hospitals, there is no professional rank or title for midwives. This shortage of midwives has been an important factor in the high percentage of caesarean section deliveries.^19^

The completeness of the maternal mortality registered in ARMCH is important to assess. Our analysis shows that over the period of 1996–2015, the maternal mortality ratio in the registry was on average about 35% lower than the national estimates in GBD 2016 and the National Office for Maternal and Child Health Surveillance of China.^20^ Given the widespread under-registration and late registration of livebirths in selected counties in China, we expected that the proportion of missing maternal mortality data in ARMCH would be greater. We know of no study that has assessed under-registration of maternal deaths in ARMCH and, therefore, we could not apply an adjustment for under-reporting to the raw ARMCH data at the county level. However, the maternal mortality ratio estimates provided in our study are bounded by the national-level estimates from GBD 2016, which means that we scaled our estimates for every district and county to the national level estimates by first adjusting livebirths then maternal deaths. Although this approach ensures that our aggregated maternal mortality ratio estimates are consistent with the GBD 2016 national-level estimate, it does assume that the same scaling factor is adequate for all counties in China. To validate this assumption, we compared our maternal mortality ratio estimates with the reported data from China's National Maternal and Child Health Surveillance System, which covered 176 counties in 1996 and 334 in 2015. We ran a simple linear regression without a constant, using county-level maternal mortality ratios from the National Maternal and Child Health Surveillance System as an independent variable and the estimated county-level maternal mortality ratio from this study as a dependent variable after excluding 11 county-years in which counties had maternal mortality ratios greater than 500 per 100 000 livebirths. The coefficient for the maternal mortality ratio from the National Maternal and Child Health Surveillance System was 1·01. showing that our findings are not biased compared with the national surveillance data (appendix).

Our analysis had the following additional caveats. First, the analysis did not include other potential sources of data on maternal mortality, such as the Disease Surveillance Point system administered by the Chinese Center for Disease Control and Prevention or the newly established Cause of Death Reporting System of the National Health Commission of China, which is a vital registration system. Such data on causes of death are likely to help triangulate more accurately levels of and trends in maternal mortality. Second, we used a Bayesian mixed-effects model to estimate directly maternal mortality ratios at the county level. Although this modelling approach accounts for structured and unstructured variation in spatial effects at the county level, it does not include information at the provincial or country levels at the same time. Future assessments of maternal mortality ratios at the county level should take advantage of information from different location hierarchies (provincial, national, or both simultaneously) and other sources, even for a subset of counties. Third, our assessment of maternal mortality ratio is not age specific due to limitations of the data from ARMCH. However, constantly decreasing total fertility rate in China in the past two decades, the resulting older age distribution of age-specific fertility, and the substantial change in the national family planning policy in China in 2015, differential trends in maternal mortality ratio by age group will be important to assess. For instance, among women aged 35 years or older, who are at increased risk of maternal mortality, initial data analysis by China's National Maternal and Child Health Surveillance System shows roughly 40% increase in the maternal mortality ratio in China from 2015 to 2016. Fourth, given the magnitude of domestic migration in China, maternal mortality might be under-reported, especially in metropolitan areas where the greatest numbers of migrants reside. Fifth, our analysis does not provide cause-specific maternal mortality ratio estimates, largely due to the constraints of the data in ARMCH. The use of multiple data sources from provincial agencies will help to estimate cause-specific maternal mortality ratios for all counties with the use of more advanced geospatial models, as have been used by Dwyer-Lindgren and colleagues.^11^ Such estimates should prove more useful in devising targeted intervention programmes at the county level in China. Sixth, we scaled our county-level estimates to the country level in GBD 2016, which provided estimates for every year covered and used multiple sources of data on maternal mortality in the estimation process. WHO also provides country-level assessments of maternal mortality ratios, including for China.^21^ Even though the maternal mortality ratio estimated by WHO for China is higher than that in GBD 2016, the overall pattern of county-level maternal mortality ratios remains the same after scaling. Lastly, both ARMCH and this analysis only included maternal mortality during pregnancy or within 42 days of birth or termination of pregnancy. However, during this period local health authorities in China usually use all possible medical and administrative resources to lower the risk of maternal mortality. Yet, there is still insufficient attention paid to late maternal mortality in China. Future research should include investigation of the proportion of late maternal deaths at the county level in China. A new national surveillance system that records data on women who came close to maternal death but survived will help this analysis.

Given the tremendous progress in lowering maternal mortality at the country and county levels in China in the past two decades, highlighting the notable heterogeneity in maternal mortality ratios between counties is important. The contrast between counties with the lowest and highest maternal mortality ratios is a reminder that much remains to be done. Even though the counties with the highest maternal mortality ratios had shown rapid decline in maternal mortality in the previous two decades (eg, annualised decrease of 7·6% in Zanda County, which was 37·1% faster than the MDG 5 target rate), maternal mortality ratios in western counties remain high. Studies focused on maternal mortality ratios in that region have pointed out that access to health-care facilities, quality of health care in rural and remote areas, and cultural practices are the most important factors associated with high maternal mortality ratios in China.^3^ In Tibet, which has the most counties with high maternal mortality ratios, 28 counties had hospital delivery rates less than 90% in 2015. Zanda County, which had the second lowest reported hospital delivery rate at 43·2%, had the highest maternal mortality ratio among all counties in China. In the current decentralised system, funds for health care are no longer proportionally distributed across counties and the local authorities are required to raise money by investment instead.^3^ For economically less developed counties, therefore, it is challenging to improve population health further.

461 counties (16·2%) of the 2852 counties in China had maternal mortality ratios at least twice as high as the national level in 2015. Our analysis provides a roadmap for targeted interventions by central and provincial governments to maintain progress in reducing maternal mortality ratios at the subnational level in the SDG era in China. Our findings also provide a great opportunity for researchers to look into what factors are associated with maternal mortality ratios and consider what intervention programmes might be the most effective and efficient to improve maternal health. More importantly, enhanced data collection with more detailed information on causes of death, the age groups of women, and late maternal mortality at the county level in ARMCH, will be necessary in the future. Concerted efforts by local health authorities, national coordination agencies, such as ARMCH and the newly established national birth registry, are needed to capture and record properly each livebirth and maternal death.
