# Supplementary material for: Maternal mortality ratios in 2852 Chinese counties, 1996–2015, and achievement of Millennium Development Goal 5 in China: a subnational analysis of the Global Burden of Disease Study 2016
Source: Lancet. 2019 Jan 19;393(10168):241–52. doi: 10.1016/S0140-6736(18)31712-4 (PMC6336935; doi:10.1016/S0140-6736(18)31712-4)

THE LANCET

Supplementary appendix

This appendix formed part of the original submission and has been peer reviewed. We post it as supplied by the authors.

Supplement to: Liang J, Li X, Kang C, et al. Maternal mortality ratios in 2852 Chinese counties, 1996–2015, and achievement of Millennium Development Goal 5 in China: a subnational analysis of the Global Burden of Disease Study 2016. *Lancet* 2018; published online Dec 13. http://dx.doi.org/10.1016/S0140-6736(18)31712-4.

APPENDIX

Appendix Table 1. Model coefficients and standard errors for fixed effects

	mean	sd
Constant	127.9132	2.6034
LDI [log scale]	-0.2326	0.0147
Maternal Education	-0.0583	0.0079
Time	-0.0667	0.0013

Appendix Figure 1. Distribution of MMR [log scale] from Annual Report on Maternal and Child Health, 1996-2015

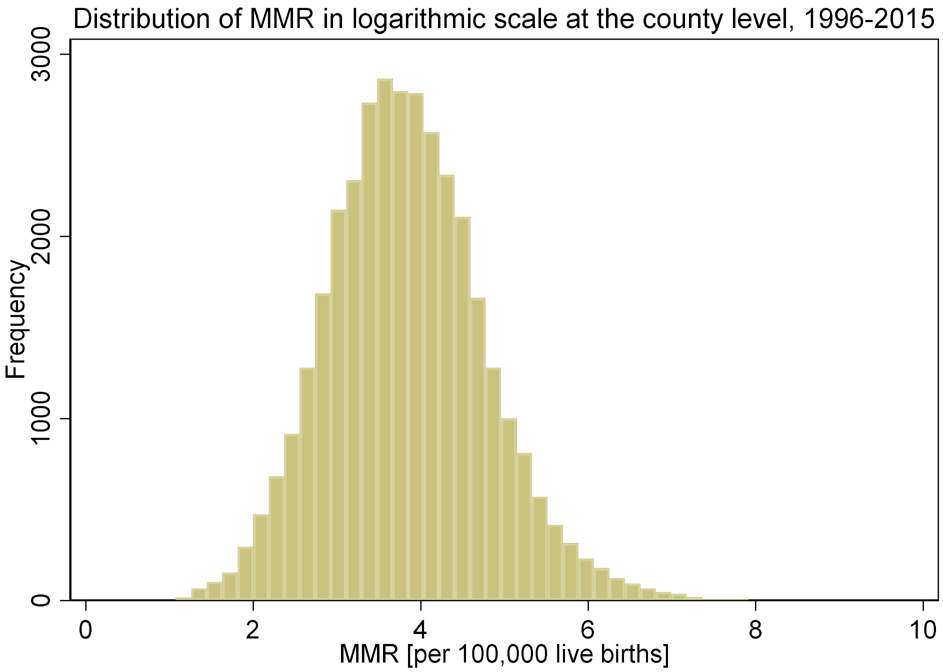

Supplement: Supplementary appendix [file mmc1.pdf]
